# Cardiomyocyte Death and Genome-Edited Stem Cell Therapy for Ischemic Heart Disease

**DOI:** 10.1007/s12015-020-10096-5

**Published:** 2021-01-25

**Authors:** Hyun-Min Cho, Je-Yoel Cho

**Affiliations:** Department of Biochemistry, BK21 PLUS Program for Creative Veterinary Science Research and Research Institute for Veterinary Science, College of Veterinary Medicine, Seoul National University, Gwanak-ro1, Gwanak-gu, Seoul, 151-742 South Korea

**Keywords:** Cardiomyocytes, Myocardial ischemia/reperfusion (I/R) injury, Cell death, Stem cells, Genome-engineering, Preclinical application

## Abstract

Massive death of cardiomyocytes is a major feature of cardiovascular diseases. Since the regenerative capacity of cardiomyocytes is limited, the regulation of their death has been receiving great attention. The cell death of cardiomyocytes is a complex mechanism that has not yet been clarified, and it is known to appear in various forms such as apoptosis, necrosis, etc. In ischemic heart disease, the apoptosis and necrosis of cardiomyocytes appear in two types of programmed forms (intrinsic and extrinsic pathways) and they account for a large portion of cell death. To repair damaged cardiomyocytes, diverse stem cell therapies have been attempted. However, despite the many positive effects, the low engraftment and survival rates have clearly limited the application of stem cells in clinical therapy. To solve these challenges, the introduction of the desired genes in stem cells can be used to enhance their capacity and improve their therapeutic efficiency. Moreover, as genome engineering technologies have advanced significantly, safer and more stable delivery of target genes and more accurate deletion of genes have become possible, which facilitates the genetic modification of stem cells. Accordingly, stem cell therapy for damaged cardiac tissue is expected to further improve. This review describes myocardial cell death, stem cell therapy for cardiac repair, and genome-editing technologies. In addition, we introduce recent stem cell therapies that incorporate genome-editing technologies in the myocardial infarction model.

**Figure:**
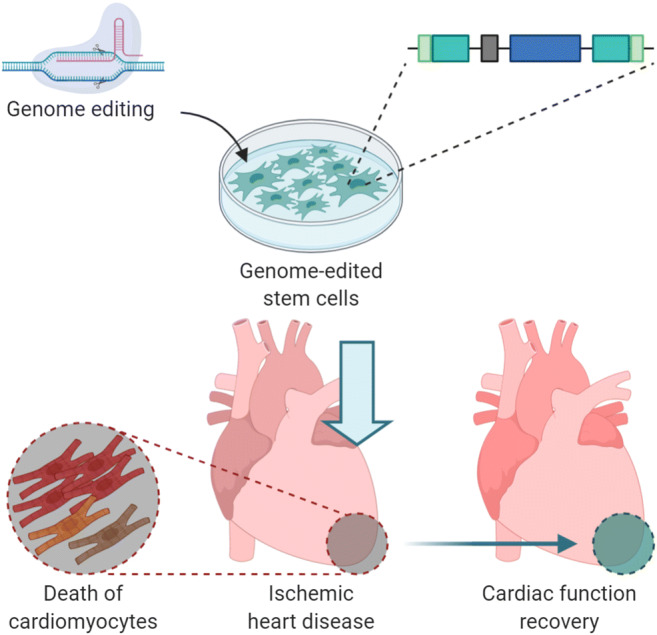
Graphical Abstract

Graphical Abstract

## Introduction

Ischemic heart disease is one of the leading causes of global mortality, and is known to contribute to ~30% of deaths over the age of 35 in the United States [1]. The pathological environment of the ischemic heart induces irreversible damage to cardiomyocytes, and prolonged exposure to it results in massive myocardial cell death and cardiac dysfunction [2]. To limit this process, reperfusion surgery is required. Paradoxically however, this surgery can cause additional reperfusion injury, leading to a spike in cardiomyocyte death [3]. Therefore, it is very important to understand the cell death that occurs during myocardial ischemia and reperfusion (I/R), and to find a strategy to limit this process.

Until the 1980s, the death of cardiomyocytes was not accurately accounted for, and it was only considered as a result of unregulated biological and chemical insults resulting from cardiovascular diseases [4]. However, as the concept of ‘programmed cell death’ emerged, active studies on each type of death were initiated [5]. The first of these described was apoptosis. Apoptosis is regulated by the mitochondria-mediated intrinsic pathway and death receptor-mediated extrinsic pathway, and Bcl-2, Caspase family and Apaf-1 are known to be involved in these mechanisms [6–8]. The process of necrosis was revealed a little later and, until the new millennium, it was thought that this only occurred accidentally. However, numerous studies commenced on the signaling that regulates necrosis revealing that necrosis is also divided into mitochondrial necrosis and death receptor-regulated necrosis [9]. However, unlike apoptosis, necrosis is regulated by RIPK3, MLKL, mPTP, etc. [10, 11]. Later, additional forms of cell death (autophagy, phagocytosis) began to be introduced, and the morphological features and mechanisms of each of these were also explained [12]. This increased understanding of myocardial cell death not only enabled access to massive cell loss in cardiovascular diseases, such as Ischemic heart disease, but also provided a better understanding of their role and mechanisms within the process and the site of injury [4, 13]. However, despite the progress of this research, there are still clear limitations to overcoming ischemic heart disease with current medical treatment due to the harsh environment within ischemic injury and the specificity of non-regenerative cardiomyocytes. In addition, reperfusion, a surgical technique, is most frequently used in the treatment of the disease. However, it can also paradoxically lead to further injury and death of myocardial cells [2]. Therefore, additional strategies for regeneration of damaged cardiomyocytes are needed.

In this regard, stem cell therapy has received much attention and numerous studies related to it have been conducted. Diverse stem cells, including pluripotent stem cells, tissue-derived mesenchymal stem cells (MSCs), and cardiac stem cells (CSCs) have been tested in preclinical experimental models [14–18]. Each of these cells have been confirmed to have effects such as protection of cardiomyocytes, acceleration of angiogenesis and support of heart tissue repair through paracrine effects. The stem cells are also expected to be able to regenerate damaged myocytes. This expectation has received a lot of attention with the prospect that c-kit+ progenitor cells could differentiate into new cardiomyocytes [19]. However, according to the mouse lineage tracing data, which were independently obtained in many studies, the possibility that endogenous c-kit+ cells contribute to the creation of new cardiomyocytes in the heart is less than 0.03%. Therefore, the probability that stem cell transplantation directly contributes to the regeneration of cardiomyocytes via c-kit+ positive cells is very low [20, 21]. In addition, despite these characteristics, the use of stem cells in clinical trials can be difficult due to ethical issues or insufficient therapeutic effects [18, 22]. Due to the limitations of stem cells, the need has arrived for new therapeutic strategies combined with other treatments. In recent years, stem cell-based gene therapy has continuously been studied, as this strategy seems to be able to improve the therapeutic ability of stem cells by transferring the target gene through in vitro transduction. For instance, the VEGF gene contributed to angiogenesis, and the Akt gene contributed to the pro-survival capacity of each cells [23, 24]. In addition, due to progressive advances in genome engineering technologies such as ZFNs, TALENs and CRISPR/Cas9 systems over the past decade, DNA modification in target loci has become easier and more efficient. As a result, this has enabled the improvement of stem cells’ therapeutic capacities [25–27].

In this review, we will introduce various myocardial cell deaths, their progression and their role in ischemic heart disease and reperfusion, and describe the stem cells applied to treat them, their treatment mechanisms, and their limitations. In addition, we describe new genome-engineering techniques, gene-edited stem cells, and introduce diverse research results that have applied these cells to animal myocardial infarction (MI) models.

## Cardiomyocyte Death

### Cell Death: Apoptosis and Necrosis

In the past, the only form of active and controllable cell death was believed to be apoptosis, and necrosis was considered a passive and unregulated form of cell death [28, 29]. However, in the twenty-first century, this was found to be incorrect, as a significant proportion of necrosis was discovered to be mediated through specific regulatory pathways. Although the pathways are different from that of apoptosis, the necrosis pathway also contains mitochondrial and death receptors. The necrosis mediated by death receptors was later named necroptosis [30, 31]. There are distinct features between apoptosis and necrosis. In apoptotic cells, shrinkage, the appearance of apoptotic bodies and phagocytosis of apoptotic bodies by surrounding macrophages are noted. Other distinct features include blebbing of cell membranes and chromatin condensation [32, 33]. However, recently it has been revealed that chromatin condensation can also be observed in necrotic cells. In the case of necrotic cells, the swelling of cells and organelles occurs due to malfunctions across the entire cell membrane, and eventually the cell structure collapses. At the tissue level, swaths typically appear on the adjacent cells; these are distinct points with independently progressing apoptosis. Moreover, necrosis is accompanied by acute or chronic inflammation. Over time, the dead cells enter the fibrosis stage, which cause permanent scarring [34].

As mentioned above, both types of death can occur either through the extrinsic death receptor associated pathway, or through the intrinsic mitochondria associated pathway. In apoptosis, caspase enzymes are activated in both pathways [35]. Caspase is a subclass of cysteine-dependent protease, which hydrolyzes the peptide bond of aspartic acid located in a specific motif and is activated as its procaspase forms converge into a specific complex. In the extrinsic pathway, the complex is formed due to the binding of the ligand to the death receptor. On the other hand, in the intrinsic pathway, cytochrome c is released into the cytoplasm due to a higher permeability of the mitochondrial outer membrane, and acts as a cofactor for apoptosome assembly [8, 36–38].

As opposed to apoptosis where caspase plays a role in both pathways, the signaling of necrosis is not contingent on a single molecule in both the extrinsic and intrinsic pathways. In the mitochondrial necrosis pathway, the calcium-dependent opening of the mitochondrial permeability transition pore (mPTP) that is located in the inner mitochondrial membrane (IMM) occurs. Normally, IMM is impermeable to small molecules and protons, which causes electrical and chemical gradients to form between the mitochondrial matrix and the inter membrane space. Since these gradients are essential for the production of ATP through mitochondrial respiration, it is very important to maintain the integrity of the IMM and subsequently maintain the normal functioning of mitochondria. During the necrosis process, the destruction of IMM and the opening of mPTP is induced [39, 40]. In contrast, in the death receptor pathway of necrosis, activation of receptor interacting protein kinase 3 (RIPK3) is an important event that induces necroptosis. RIPK3 is activated by homologous RIPK1 phosphorylation. Subsequently, RIPK3 phosphorylates a pseudo-kinase called mixed lineage kinase-like domain (MLKL), which increases cell membrane permeability and finally induces the extrinsic necrosis [41, 42].

### Apoptosis and Necrosis in Ischemic Heart Disease

**Fig. 1:**
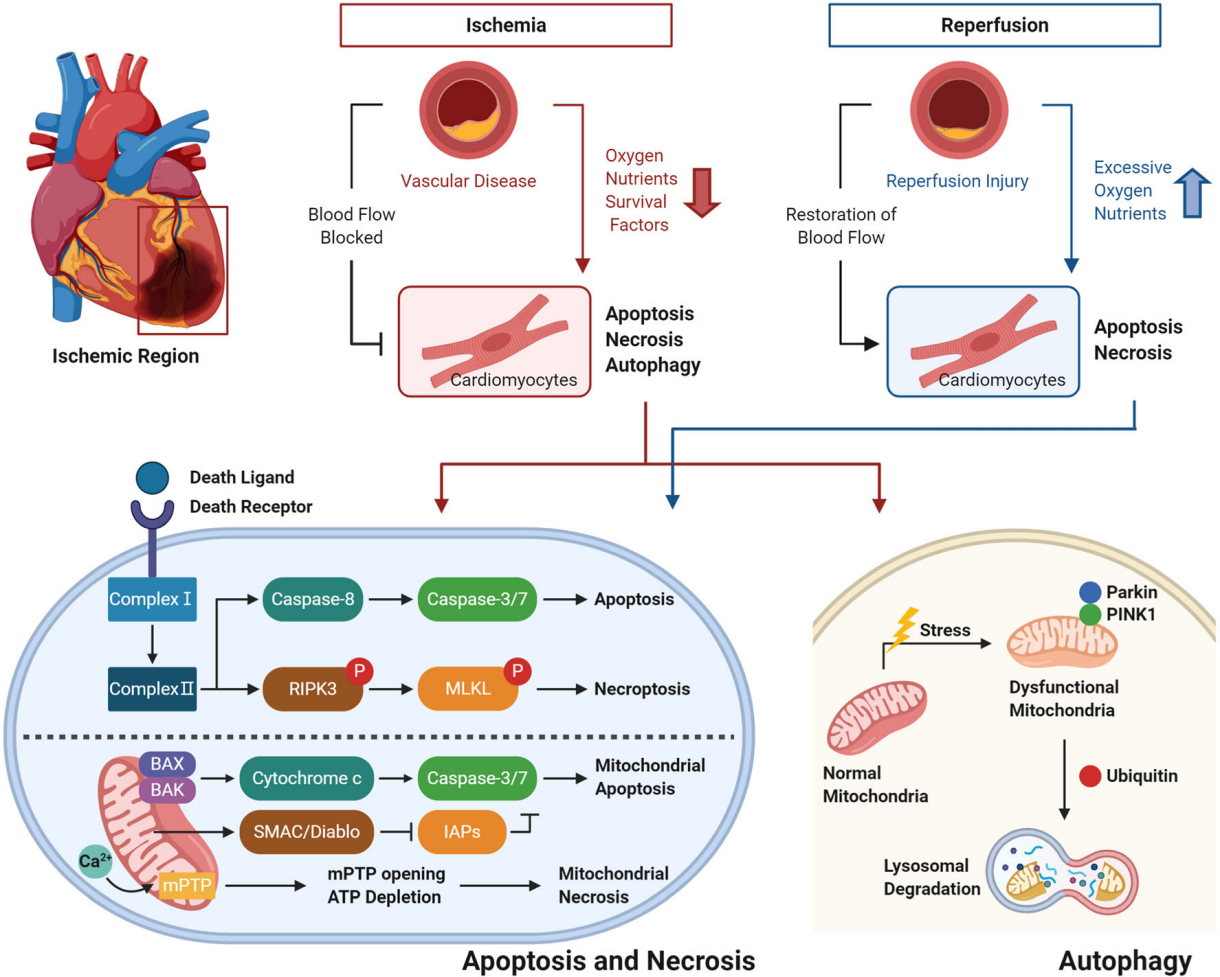
Overall path of cardiomyocyte death in ischemic heart disease and reperfusion. The ischemic heart environment and reperfusion induce myocardial cell death. Of these cell deaths, both apoptosis and necrosis are mediated by death receptors and mitochondria. These are divided into death receptor-mediated apoptosis and necroptosis, and mitochondria-mediated apoptosis and mitochondria-mediated necrosis, respectively. Both types of apoptosis involve the activation of Caspase-3/7, while the necroptosis is caused by permealization of the plasma membrane due to the activation of MLKL. Mitochondria-mediated necrosis is linked to the opening of mPTPs. In addition, an accumulation of damage to mitochondria occurs in ischemic heart disease, and it causes autophagy. The process proceeds as Parkin accumulates and PINK1 is transferred to the inside of the mitochondria

In ischemic heart disease, cardiomyocytes do not receive adequate amounts of oxygen, nutrients, and survival factors. The cause of this disease is thrombotic occlusion in the coronary artery due to rupture of the atherosclerotic plaque [43]. The ischemia induces various structural and functional changes in the heart, the most prominent of which is the death of cardiomyocytes. To prevent further progression of the disease, reperfusion therapy is essential [2]. Since this process can re-supply blood and oxygen to the heart tissue, the effectiveness of the therapy is unquestionable. However, the reperfusion process also paradoxically causes the death of cardiomyocytes at a quantity comparable to ischemic injury [44]. The main deaths that occur in myocardial ischemia and reperfusion are apoptosis, necrosis and autophagy [13]. To overcome the death of cardiomyocytes and advance heart disease treatment, it is necessary to understand the process and mechanism of these deaths (Fig. 1).

### Mitochondrial Pathways of Cardiomyocyte Death

Early studies speculated that apoptosis and necrosis were mitochondrial-induced cell deaths following myocardial I/R, and recent studies have proved that the mitochondria indeed play a central role in both pathways. As previously explained, when myocardial ischemia progresses, the oxygen and nutrients begin to deplete rapidly. In this harsh ischemic environment, ROS production, cessation of mitochondrial respiration, and the activation of anaerobic metabolism are induced [13, 45]. The mitochondria are the main target of damage induced by ROS. In myocardial I/R, mitochondrial apoptosis begins according to these procedures: mitochondrial swelling, rupture of the outer membrane, and releasing of pro-apoptotic factors (E.g. cytochrome c, SMAC/Diablo) [2, 46]. In addition, an enhanced release of cytochrome c and MOMP due to the opening of mPTP induced by oxidative stress and overload of [Ca^2+^]can in turn induce the apoptosis of cardiomyocytes [47]. The harsh environment also directly affects the mitochondrial necrosis of cardiomyocytes. Ischemia activates the anaerobic metabolism, which causes intracellular acidosis. To control the acidosis, cells release [H^+^] through the Na^+^/H^+^ exchanger. However, this process results in an increase of [Na^+^] in the cell. Then in handling the excessively high [Na^+^], the Na^+^/Ca^2+^ exchanger increases the intracellular [Ca^2+^] level. Ultimately, these events contribute to the opening of mPTPs, which is directly related to the necrosis of cardiomyocytes [48, 49].

As described above, the process of reperfusion can also activate the death of cardiomyocytes induced by mitochondria. The sudden re-supply of oxygen and ATP by reperfusion acts as a double-edged sword on the necrosis of cardiomyocytes [50]. For mitochondria, the supply of ATP is not only essential for maintaining mitochondrial potential and sarcolemmal ion gradients, but also reactivates sarcoplasmic reticulum ATPASE 2A(SERCA2A), which pumps Ca^2+^ into the SR. However, hyperactivation of the SR Ca^2+^ release channel results in faster SR Ca^2+^ uptake and release cycles. In this way, the level of ATP is restored, yet the overloaded state of cytosolic Ca^2+^ causes the cardiomyocytes to become hypercontracted. This condition can be observed for the first few minutes after reperfusion, which appears as ‘contraction band necrosis’ in histological analysis. Excessive release of SR Ca^2+^ causes mitochondria to take up Ca^2+^ through the mitochondrial calcium uniporter (MCU). The mitochondrial Ca^2+^ overload resulting from this process induces the opening of mPTP and prevents the mitochondria from maintaining the mitochondrial membrane potential (ΔΨm), and eventually this progresses to necrosis [51, 52].

### Death Receptor Pathways of Cardiomyocyte Death

To investigate the role of Fas signaling after myocardial I/R, Fas deficient mice were designed. The reduction of infarct size and TUNEL positive cardiomyocytes were confirmed in this model. The decrease in TUNEL positive cells indicated a reduction of apoptosis, and Fas signaling was demonstrated to induce the apoptosis of cardiomyocytes, which causes serious damage to the myocardium [53]. Similar research was conducted in RIPK3 knock out mice. In a myocardial I/R model, the deficiency of RIPK3 reduced the infarct size. In this regard, Ca/calmodulin-dependent protein kinase IId (CaMKIId), an accelerator of mPTP-mediated necrosis, has been identified as a target for RIPK3. It was also confirmed that RIPK3 activates CaMKIId through phosphorylation. Moreover, since CaMKIId is known as a mediator of mPTP opening and by extent I/R damage, the pharmacological inhibition of this kinase can prevent mPTP opening and the loss of cardiomyocytes [54, 55]. From this information, the necrosis of cardiomyocytes mediated by the opening of mPTP can be inferred.

### Cell Death: Autophagy

Autophagy is the mechanism by which cells decompose components within themselves. This death is closely related to the stress-induced pathway, and the formation of massive vesicles by lysosomes is a major feature of it [56]. The vesicles separate organelles and protein aggregates from the rest of the cell. Autophagy recycles the elements that make up the cells, contributing to the maintenance of nutrition and energy homeostasis. The stresses that induce this type of cell death are starvation, oxidative stress, I/R environment, etc. [57]. Autophagy begins with the formation of the isolation membrane, which consists of a double membrane shaped like a cup. This isolation membrane originates from different structures depending on the stimulus. When these stimuli persist, the end of the membrane wraps around, forming an autophagosome, which eventually results in the separation of cellular components and organelles. The autophagy that removes abnormal mitochondria is of particular interest and is called mitophagy. In addition, proteins such as Parkin, PINK1, BNIP3, NIX, etc. are known to act specifically in mitophagy [56–58].

### Autophagy in Ischemic Heart Disease

Accumulated damage to the mitochondria of cardiomyocytes in the ischemic region leads to an overall decrease in cell function and the formation of an inflammasome. Therefore, it is important to remove the toxic contents and maintain the quality of mitochondria [58, 59]. The well-established mechanism of autophagy in cardiomyocytes involves the accumulation of PINK1 (PTEN-induced putative kinase-1). When the outer membrane of the damaged mitochondria is depolarized, the E3 ubiquitin ligase, Parkin, is brought into the mitochondria. Parkin introduced in this way is activated through phosphorylation by PINK1. These processes eventually lead to autophagy in cardiomyocytes [60].

### Stem Cell-Based Therapy for Cardiac Repair

**Fig. 2:**
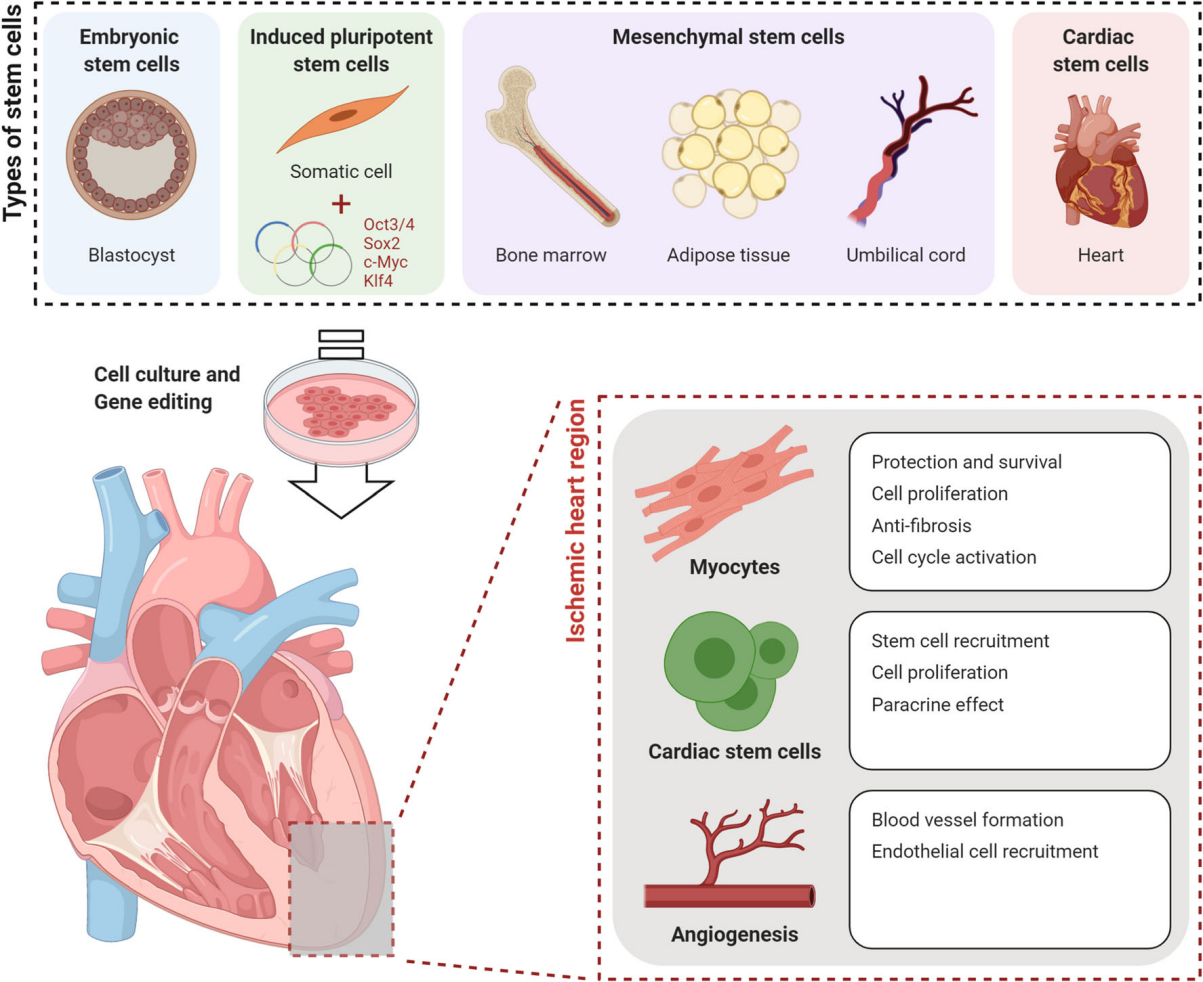
Types of stem cells applied for ischemic heart disease and their therapeutic effects on damaged heart. Various stem cells, including embryonic stem cells, induced pluripotent stem cells, mesenchymal stem cells and cardiac stem cells are investigated for the treatment of ischemic heart disease. Each cell has different capacities for therapeutic effects. The main purpose of the application of these cells is the protection and regeneration of cardiomyocytes, cardiac stem cell recruitment and promotion of angiogenesis

Myocardial I/R causes massive cell death of cardiomyocytes, due to apoptosis, necrosis, necroptosis, autophagy, etc. To date, known therapies related to this, such as coronary intervention or a surgical approach, have been very limited. Since cardiomyocytes are fully differentiated cells, they have a limited regenerative ability [61]. Therefore, it is almost impossible to regenerate damaged cardiomyocytes with the above-mentioned methods alone. Moreover, as described, the reperfusion process induces the additional death of cardiomyocyte. Thus, an additional alternative is required. With regard to these additional therapies, the most studied avenue is the regeneration of the lost cardiomyocytes through stem cell therapy. In the following sections, the types of cells used in cardiovascular disease, their therapeutic effects and their mechanisms will be explained (Fig. 2), and additionally the limitation of each stem cell will be discussed.

### Pluripotent Stem Cells

Pluripotent stem cells have the capacity of self-regeneration and can differentiate into any type of cell in the whole body. Of these, ES cells are derived from the inner cell mass of blastocysts, and have the capacity to develop up to three germ layers [62]. ESCs can also differentiate into contracting cardiomyocytes. And the feature made the expectation that the application of ESC-derived cells could regenerate damaged myocardium in their application. Numerous studies have been conducted to advance the efficiency of differentiation from ESCs to cardiomyocytes. But currently, it is impossible to produce mass amount of terminally differentiated cardiomyocytes from the ESCs in vitro. The therapeutic application of these cells is vulnerable due to additional difficulties and has shown its therapeutic limitations. First of all, hESCs are always accompanied by ethical and political issues surrounding the process of obtaining these cells from humans [63]. In addition, pluripotency, the competence of ESCs, paradoxically induces the potential risk of teratoma formation. Although, the probability is even lower, the differentiated cells derived from ESCs also carry a risk of teratoma formation, as massive amounts of cells are required for cell therapy of MI [64].

Delivery of human ESC-derived cardiomyocytes to the myocardium of rat MI models resulted in diminished LV remodeling, and assisted restoration of heart function. In non-human primates, similar therapeutic effects have been confirmed, like enhancement of LVEF, and improved heart tissue repair. However, the risk of hESC-derived cardiomyocytes and the therapeutic limitation of these cells were also revealed in this study. Liu et al. injected 750 million hESC-CMs intramyocardially into four pigtail macaques. Arrhythmias occurred in one of the 5 cells treated group, implying that similar phenomena may also occur in humans. Four weeks after cell transplantation, the left ventricular ejection fraction increased by 2.5% in the control group, but increased by 10.6% in the hESC-CMs transplanted group [65]. The numerical value is certainly remarkable, but it is the result of implanting the huge amount of 750 million cells. This is equivalent to about 6.750 billion cells in an average 75 kg adult, while it is considered that the average 75 kg human LV contains 5 billion CMs, so it is impossible to inject this large number of cells clinically. From the results, it is unclear whether the transplantation of hESC-CMs would produce similar results when applied clinically. Therefore, the results of this study not only exhibited the potential risk of arrhythmias, but also the number of cells required to show therapeutic effects are not clinically applicable [66].

These limitations provoked the emergence of another pluripotent stem cells, induced pluripotent stem cells (iPSCs). The iPSCs are produced through reprogramming of fibroblasts using 4 factors, such as Oct3/4, Sox2, c-Myc, and Klf4 [67]. These cells have the advantages of ES cells while avoiding the ethical dilemmas. In particular, iPSCs have shown stable integration after transplantation and its regenerative capacity has been demonstrated in many studies. Furthermore, protocols that induce differentiation into various types of cells related to cardiac tissue, including cardiomyocytes, are also well established [68]. Therefore, the application of these cells in cardiovascular disease is seriously considered, and the therapeutic effects of these cells have been shown in various animal models. Experiments in which iPSC derived myocytes were injected into a rat cardiovascular disease model were conducted and successful transplantation and differentiation into myocardial cells was confirmed. It was also confirmed that the ejection fraction was recovered functionally and fibrosis was reduced [69]. In addition, experiments in which human iPSC derived cardiomyocytes were transplanted into the heart of a swine model, also confirmed various positive effects, such as improved left ventricular function and reduced infarct size [70]. However, sufficient reproducibility has not yet been secured, and a more standardized transplantation method is required to utilize these cells for therapeutic purposes. Even though there is positive data in preclinical studies and technical advances in the generation of iPSCs, there still remains many concerns. The major hinderances to clinical application are the potential of teratoma formation and immunogenicity. As the four transcription factors used to produce iPSCs are identified as oncogenes, iPSCs also contain the risk of teratoma formation. In addition, the epigenetic memory of somatic cells, unusual methylation patterns and genetic mutations that may occur during reprogramming and cell incubation, contribute to genetic instability in iPSCs. Furthermore, the immunogenicity of iPSC-derived cells is important for the clinical application [71]. Theoretically, since the iPSCs are produced using the patient’s own cells, there should be no risk of rejection after transplantation. However, there have been reported cases of immune rejection following the implantation of iPSC-derived cells. This suggests that in vitro procedures for the generation and differentiation of iPSCs may affect immunogenicity. The medical application of hiPSC-based therapy not only requires quality and safety control, but also overcoming the risk of teratoma formation and consideration of the immunogenicity [72].

### Mesenchymal Stem Cells

Mesenchymal stem cells (MSCs) are a type of adult stem cell that can be derived from various tissues such as bone marrow [73], adipose tissue [74] and umbilical cord blood [75]. Each of these MSCs have different isolation protocols and culture conditions, and due to their various sources, each cell type has their own unique properties. Since they are multi-potent stem cells, they could differentiate into various cells and they also have the capacity to renew themselves [76]. Although there is no clear standard for the definition of MSCs yet, the classification criteria for MSCs specified by the International Society for Cellular Therapy are as follows: These cells should express different sets of clusters of CD markers, such as CD90, CD105, and CD73. In contrast, hematopoietic lineage CD markers such as CD45, CD34, CD14, and CD19 should be absent from expression [77]. MSCs are considered to be good candidates for cell therapy because they are relatively simple to isolate and secrete various substances that can activate tissue repair. Furthermore, these stem cells modulate the immune responses, promote angiogenesis and exert antioxidant effects through the secretion of paracrine factors [76]. These features ensure sufficient stability for MSCs to be used for therapeutic purposes in cardiomyopathy patients. The most studied MSCs are those derived from bone marrow (BM-MSCs) and adipose tissue (AT-MSCs). These cells are easily isolated from each individual tissue, and have the advantage of being able to be produced in large amounts while maintaining their properties. However, these cells have the disadvantage of not having identical properties after isolation. Recently, human umbilical cord blood derived mesenchymal stem cells (hUCB-MSCs) have received special attention in regenerative medicine because they are considered to encompass both the advantages of embryonic stem cells as well as adult stem cells. Compared to adult stem cells, hUCB-MSCs have an enhanced capacity for cell proliferation and differentiation. On the other hand, compared to ESCs, these cells have a lower immunogenicity and lower potential of teratoma formation, thus enabling safer treatment [78].

The major benefit of MSC therapy is derived from the paracrine effects. The MSC secretome has cardiovascular protection effects and is known to modulate the death of residue cells caused by the pathological environment [17, 76]. Nevertheless, even though MSCs show sufficient therapeutic potential in regeneration of damaged cardiomyocytes and ischemic heart diseases, there are still obvious limitations that should be overcome, including an incomplete understanding of the therapeutic mechanism, and low settlement and survival rates of the transplanted cells [79].

### Cardiac Stem Cells

For a long time, myocytes have been considered non-renewable cells. However, there have recently been reports of possible division of myocytes, and seeing as the existence of cardiac stem cells (CSCs) in the heart has also been confirmed, interest in these cells have increased [80]. CSCs are not only capable of differentiation into cardiomyocytes, smooth muscle cells, and endothelial cells, but also have expressed markers related to mesenchymal stem cells (CD90, CD105), embryonic stem cells (Nanog, Sox2), as well as early cardiogenesis markers [81, 82]. CSCs can be subdivided into c-kit+ cells, Isl-1+ cells, cardiac mesoangioblasts, and cardiosphere-derived cells (CDCs), depending on the markers expressed on their surface. These cells are known to express more cardiovascular markers than MSCs, and it was also confirmed during in vivo experiments that these cells derived from myocardial tissue could differentiate well into cardiomyocytes when injected into the heart of a rat MI model [83]. However, additional ex vivo studies are required to prove the effectiveness of CSCs and explain their process in more detail. In addition, research using growth factors, miRNA, etc., which can further enhance the capabilities of these cells, should be continuously conducted.

### Cardiosphere-Derived Cells (CDCs)

Cardiosphere-derived cells (CDCs) are a type of cardiac progenitor cells (CPCs) that is composed of heterogeneous cells. These cells originate from the heart tissue, and form as an intermediate step through the cardiospheres, self-assembled multicellular clusters. CDCs are clongenic and have the potential to differentiate into three cell types that make up the heart: cardiomyocytes, endothelial cells, and smooth muscle cells, thus they fulfill the core criteria as cardiac stem cells. Since the regeneration rate of cardiomyocytes is only about 1 ~ 2% in adult humans, CDCs that can differentiate into cardiac lineages are good candidates for the treatment of MI [84].

The injection of human CDCs in rat MI models, not only reduced the infarct size, but also proved their therapeutic effect on the left ventricular injection fraction (LVEF). Since CDCs can differentiate into three major heart cell types, it was expected that the results would directly affect cardiac regeneration. However, it was proved that the main effects are indirect paracrine effects. In another preclinical research study using small preclinical models, administration of CDCs alleviated inflammation, reduced the fibrosis, and diminished the apoptosis caused by oxidative stress [85, 86].

In mini-pig models of MI, intracoronary injection of autologous CDCs exhibited a reduction of infarct size, alleviation of LV remodeling, and an improvement of the hemodynamic indexes [87]. Furthermore, another study confirmed the stability and therapeutic efficiency of intramyocardial injection of autologous CDCs and was also conducted in the pig models of MI. The study optimized the cell dose of CDCs (0.5 million cells/site), that are injected into the target tissue (20 peri-infarct sites) and compared the therapeutic effects with cardiospheres, which is the precursors of these cells. In 8 weeks of observation, the cardiospheres-treated groups showed little change in LVEF, compared to a 7% reduction in the placebo-treated groups. And the CDCs-treated groups show an increase of ~3% in LVEF. This data indicates that the CDCs and cardiospheres mitigate the deterioration of LVEF due to MI [88, 89].

### Cardiac Mesenchymal Cells (CMCs)

According to several studies, c-kit+ CPCs have similar characteristics with mesenchymal stem cells, and express MSC markers CD90, CD105, and CD73. In addition, according to the results of preclinical studies with mice, rats, and pigs, treatment with c-kit+ CPCs showed heart function recovery and also alleviated cardiac remodeling. Despite these positive results, the main obstacle to the use of c-kit+ CPCs is the instability of c-kit expression during in vitro culture. For example, the murine c-kit+ CPCs showed a decrease in c-kit expression from 78% to less than 30%, after 4 culture passages [90, 91].

However, it has been reported that myocardial cells capable of adhesion to plastic can be sorted to cell populations that maintain prominent c-kit expression. After cardiac digestion, the cells are divided into two populations: rapidly adhering (RA) cells that adhere in <24 h and slowly adhering (SA) cells that only adheres after 24 h. These cell populations exhibit similar phenotypes with mesenchymal stem cells, and were thus termed as cardiac mesenchymal stem cells (CMCs). SA c-kit+ CMCs and RA c-kit+ CMCs were respectively transplanted into infarcted mouse heart and their beneficial effects were evaluated. SA c-kit+ CMCs showed preservation of heart function and reduced fibrosis, whereas RA c-kit+ CMCs did not. A similar result was confirmed in vascularization; SA c-kit+ CMCs, but not RA c-kit+ CMCs, showed increased vascular density after transplantation [90, 92].

A study was conducted to isolate CMCs with adherence to plastic alone without c-kit sorting [93]. In this study, rapidly adhering (RA) and slowly adhering (SA) CMCs were isolated from mouse hearts and expanded in vitro. Afterwards, these cells were transplanted into a mouse MI model, and the treatment effect was evaluated. On the day 28, the vehicle-treated mice group did not show a significant difference with RA CMC group. But the SA CMC group showed a significant improvement result (about 7% increase in LVEF). In histological analysis, collagen deposition was decreased only in the SA CMCs group. However, new cardiomyocytes or angiogenesis were not identified in both SA and RA groups. Therefore, the major therapeutic mechanism of SA CMCs was considered to be due to paracrine effects in this study [93]. Additionally, as the number of CD45+ cells decreased in the SA but not in RA CMCs group. It suggests that SA CMCs also have anti-inflammatory effects, which could confirm the result of fibrosis attenuation. This study, based on a simpler method of adhesion to plastic, made it easier and less expensive to isolate CMCs. By omitting the sorting step, it is meaningful in that it enabled the production of cells as younger passages as clinically applicable [94].

### Limitations of Stem Cell Therapy

The therapeutic effects of stem cell therapy in cardiovascular disease has been demonstrated not only in the various studies, but also in preclinical models where positive results have been reported [2, 83]. However, there are apparent limitations that still need to be solved in order to utilize stem cell therapy in patients with cardiovascular disease [17]. In this regard, the introduction of additional genes reinforces the function of stem cells while still retaining the advantages of existing stem cell therapy. Besides, unlike other strategies, there is no need to acquire new resources and there is no concern about any additional effects on the stem cells. Therefore, the genetical enhancement of stem cells has attracted much attention as a promising new direction for stem cell therapy.

### Genome-Edited Stem Cells: Therapeutic Application in Myocardial Infarction Models

#### Genetically Modified Stem Cells Via Viral Infection

Conventionally, the technique used for genetic modification of stem cells was lenti-viral infection for gene insertion and deletion. Lentivirus is a retrovirus with a single-stranded RNA genome, and it has a reverse transcriptase to transduce genetic elements in cells [95, 96]. Many genes were introduced into various stem cells through viral infection, and the therapeutic effects and cardiomyocytes protective effect when transplanted into the cardiovascular disease models were confirmed (Table 1). Akt (protein kinase B) 1 has a pro-survival capacity, and when depleted in an Akt knock out mouse (Akt −/−) an abnormal proliferation of cardiomyocytes is noted, which indicates fatal heart defects [97]. When the Akt1 gene was introduced into mouse CPCs, an improved proliferation capacity was confirmed. Furthermore, an additional secretion of various positive factors was also confirmed, and when transplanted into a mouse MI model, these cells enhanced the mobilization of endogenous c-kit+ cells [98]. These therapeutic effects are also confirmed in human bone marrow-derived MSCs [99]. SDF-1, a chemokine that activates STAT3 and Akt signaling, is also involved in the survival and homing of progenitor cells. The expression of this gene increases after ischemic heart injury, and it is known to be needed to repair heart tissue. However, this increase in expression is considered temporary and insufficient [100, 101]. In a case of MSCs that additionally delivered the SDF-1 gene, when they were injected into the rat ischemic heart disease model, they showed a 240% improvement in cardiac function over the control group [102]. Furthermore, MSCs that overexpressed CXCR4, which is the receptor of SDF-1, also showed additional recovery effects when transplanted into the rat ischemic heart disease model. The genetic modification of SDF-1/CXCR4 enhanced the therapeutic effects in ischemic heart injuries through various mechanisms. These mechanisms are, respectively, the recruitment of CSCs, and the attraction of CXCR4+ endothelial progenitor cells, which induce angiogenesis through VEGF signaling and additionally induce secretion of various paracrine factors related to cell proliferation, survival and engraftment [103].

**Table 1:** Recent studies of combined stem cell and gene therapy for ischemic heart disease

**Cells type**	**Genes**	**Transfection Methods**	**Ref**
Mouse Cardiac Progenitor Cells	Akt (+)	Renti-virus infection	[98]
Human Bone Marrow-derived Stem Cells	Akt (+)	Retro-virus infection	[99]
Human Mesenchymal Stem Cells	SDF-1 (+)	Retro-virus infection	[102]
Rat Mesenchymal Stem Cells	CXCR4 (+)	Retro-virus infection	[103]
**Cells type**	**Genes**	**GE Techniques**	**Ref**
Human Umbilical Cord Blood-derived Mesenchymal Stem Cells	VEGF (+)	TALEN	[126]
Human Umbilical Cord Blood-derived Mesenchymal Stem Cells	HGF (+)	TALEN	[127]
Human Embryonic Stem Cells	MESP1(+)	TALEN	[129]
Human Umbilical Cord Blood-derived Mesenchymal Stem Cells	LEF1 (+)	CRISPR/Cas9	[132]
rhesus macaque induced pluripotent stem cells	NIS (+)	CRISPR/Cas9	[133]
Human Umbilical Cord Blood-derived Mesenchymal Stem Cells	B2M (−)	CRISPR/Cas9	[136]
Amniotic Mesenchymal Stem Cells	IL-10 (+)	TALEN	[137]
Bone Marrow-derived Mesenchymal Stem Cells	IL-10 (+)	CRISPR/Cas9	[138]

### New Genome-Editing Technology

Combining stem cell therapy with gene therapy through the integration of therapeutic genes improves its therapeutic function, and it is thus emerging as a new therapeutic strategy that can confront incurable diseases such as ischemic heart disease [104, 105]. Various gene combined stem cell therapies are being studied to treat the myocardial infarction model. However, the method used most in these studies to introduce the target genes is transfection using retro or lenti viral vectors. This method has been utilized until recently due to its various advantages, such as high efficiency and persistence of expression [106]. However, it does elicit some serious concerns such as the possibility of abnormal cellular transformation due to random integration, tumor genesis, etc. [107]. Therefore, the emergence of new gene-editing technologies that can overcome these limitations has been required.

New genome editing strategies have emerged that enable DNA modifications, such as gene insertion or deletion, in cells and living organisms [108]. In order for these technologies to be successfully applied to clinical treatments using stem cells, sufficient efficiency and stability must first be secured, and there should also not be any unintended physiological effects on the targeted cells. For this to be technically feasible, specific endonucleases (SENs) are required to cleave the target site [109]. When these endonucleases cause DNA distortions, the cells begin repairing the DNA with breaks through two different mechanisms: non-homologous end joining (NHEJ) or homology-directed repair (HDR). Of these, NHEJ occurs more frequently and progresses rapidly, and it can be used to cause small insertions or deletions (indels) on the gene. On the other hand, an additional donor vector is required in the case of HDR. After a double strand break, sequences of donor vectors compatible with the site are incorporated into the endogenous locus, allowing for precise genetic modification [109]. As such, genome editing technologies operate based on site specific endonucleases, which include zinc finger nucleases (ZFNs), transcription activator-like effectors nucleases (TALENs), and clustered regularly interspaced short palindromic repeat (CRISPR)/CRISPR-associated system 9 (CRISPR-Cas9) systems [110].

### ZFNs, TALENs, CRISPR/Cas9

ZFNs were the first specific endonucleases utilized for genome editing. This nuclease is composed of two parts, a DNA-binding domain and a DNA-cleavage domain derived from the FokI restriction enzyme. The DNA binding domain can bind to a DNA sequence of up to 18 bp, and the target site of ZFN consists of a guanine-rich 3 bp subsite expressed by 5′-GNN-3′ [111]. When this domain recognizes and binds to the genome, the cleavage domain can cleave the DNA, thereby enabling site-specific genome engineering [112]. However, the design of zinc finger domains for these ZFNs is difficult and the GE efficiency is low, so it shows clear limitations for practical clinical applications [113]. Due to the identification of the DNA recognition protein possessed by bacteria, TALEN, the next gene editing technology, was developed. TALEN is composed of a FokI nuclease domain on the C-terminal side and a DNA-binding TALE-repeat domain on the A-terminal side [114]. ZFNs and TALENs have in common that they both have a FokI endonuclease domain and two DNA bindings are required in a remote region to activate the cleavage process. The DNA binding domain of TALEN contains a residue of a repeat sequence consisting of ~34 amino acids, and DNA cleavage occurs between ~12 and 20 bp around the spacer sequence. Unlike ZFNs, TALENs have the advantage of being theoretically able to edit any DNA sequence, as well as being more efficient and less toxic in its genome editing [113].

CRISPR was first discovered in *E. coli* in 1987 in the form of a short tandem repeat sequence [115]. Later, in 2013, gene editing technology using it was first introduced, and since then this technology has been continuously developed [116]. CRISPR/Cas9 consists of a Cas9 nuclease and a single guide RNA. The single guide RNA consists of crRNA and tracrRNA that can specifically recognize DNA sequences, and which are bound to Cas9 with CRISPR RNA. Compared to other technologies, the target site is not remote, and it is very easy and simple to design, and since it shows relatively high specificity and efficiency, many people are attempting gene editing using the CRISPR/Cas9 system. In addition, it has a lower cytotoxicity than ZFNs, and because it is capable of multiple gene mutations, it has a higher preference than other gene editing techniques [117, 118].

### Therapeutic Application of Genome-Engineering Systems to Induced Pluripotent Stem Cells

Many studies have endeavored to generate iPSCs utilizing TALENs and CRISPR/Cas9 systems [119, 120]. The iPSCs produced through genome engineering can differentiate into cardiomyocytes, that can be delivered to the infarcted heart for treatment. The iPSCs generated through TALEN and CRISPR/Cas9 systems have almost no off-target mutation problems, and have reduced the risk and limitations of iPSCs that are produced through viral infection [121]. As described above, transplantation of iPSC-derived CMs that are produced by conventional methods to infarcted heart still comes with numerous safety concerns, such as immunogenicity, teratoma formation, and the risk of arrhythmias [71, 72]. The application of genome-editing technology not only reduces the risk of teratoma formation, but also enables more stable treatment by integrating pluripotent genes such as Oct3/4, Sox2, c-Myc, and Klf4 into safe harbor sites [120]. In addition, these technologies can be used to integrate inducible-suicide genes into the iPSC genomes. If a teratoma is generated from the delivered cells, it is possible to immediately eliminate only these cells [122]. The combination of these technologies in iPSCs can resolves the risk of teratoma formation, which is the major obstacle in iPSC-CMs treatment.

### Preclinical Application of Gene-Engineered Stem Cells through the TALEN System

VEGF is a signal protein known to induce blood vessel formation [123]. When MSCs overexpressing VEGF were transplanted into a rat MI model, angiogenesis was promoted and the cell apoptosis induced by hypoxic stress was reduced [124]. This indicates that integration of the VEGF gene into MSCs can be a sufficient strategy. Despite these significant effects, gene transfer through viral infection not only prevents clinical trials from being attempted, but also carries the risk of inducing unintentional transformation [125]. In addition, excessive expression of this gene has the potential risk of oncogene activation [123]. Thus, overexpression of genes utilizing genome engineering may be a safer alternative. In research incorporating this strategy into hUCB-MSCs, TALEN has been used to integrate this gene into the AAVS1 locus safe harbor site located on human chromosome 19q, and a Tet-on system aimed at regulating the secretion of VEGF upon treatment with doxycycline has been added. This strategy not only overcomes the existing limitations, but its efficiency and regulation have also been confirmed in a rat MI model [126]. In addition, an attempt to produce MSCs that can regulate HGF secretion with a similar approach was also successfully performed. Induction of HGF in hUCB-MSCs, which is known to inhibit apoptosis of epithelial cells and play an important role in proliferation, not only enhanced the cell migration but also improved viability in harsh conditions. Inducible HGF-secreting hUCB-MSCs were applied to the mouse hindlimb ischemia model. After transplantation into the disease model, in the group in which HGF secretion was induced by doxycycline treatment, blood vessel formation was promoted and blood flow was also restored [127]. Although, it has not been verified in cardiovascular disease, the therapeutic potential is promising based on these effects.

Based on these achievements, a stent coated with inducible VEGF/HGF-secreting hUCB-MSCs was constructed. This study not only proposed a novel therapeutic approach through the combination of two different types of cells with the stents, but also confirmed their adhesion to the stent and an optimized cell ratio. In a swine model, administration of this stent to the coronary artery and the induction of VEGF and HGF with doxycycline not only reduced stent restenosis, but also promoted endothelialization. Although this study did not verify the therapeutic effect in a model that caused cardiovascular disease, this study is significant in that it demonstrates its effectiveness in the coronary array of large animal models, suggesting a stent therapy coated with genetically-engineered stem cells can be used directly for clinical treatment [128].

In addition to these, research was also conducted that induced MESP1 in an hESC line utilizing TALEN-mediated knock-in. MESP1 is transiently expressed in the early stages of cardiac development, and is known as a major transcription factor leading to cardiac progenitor cells. The induction of MESP1 in hESCs inhibited canonical Wnt signaling and promoted differentiation into cardiomyocytes and endothelial cells. Furthermore, the application of these cells to the rat MI model resulted in the prevention of cardiomyocyte death, enhancement of angiogenesis in the infarcted region, reduction of fibrosis, and finally the preservation of cardiac function [129].

### Preclinical Application of Gene-Engineered Stem Cells through the CRISPR/Cas9 System

LEF1 is a transcription factor involved in the canonical Wnt pathway [130]. LEF1 not only improves the proliferation of stem cells, but according to recent studies, it is also reported to play a major role in heart development [131]. In in vitro experiments, transduction of the LEF1 gene in hUCB-MSCs increased the rate of cell proliferation and reduced the apoptosis in harsh environments. In the rat MI model, the transplantation of LEF1 overexpressed hUCB-MSCs with the CRISPR/Cas9 technology, sustained various therapeutic effects of MSCs including paracrine effects due to improved cell survival in the infarcted area. In addition, due to improved vascularization and cardio-protection, it eventually protected heart function [132].

In stem cell therapy, consideration of cell tracking techniques for confirming the engraftment and localization of transplanted cells is essential for optimizing the treatment method and for clinical translation. However, imaging strategies capable of monitoring transplanted cells over a long-term in a non-invasive method are still lacking, and studies in animal models have not been performed sufficiently. To meet this purpose, a research was conducted in which the sodium/iodide symporter (NIS) gene was integrated into rhesus macaque induced pluripotent stem cells (RhiPSCs) through the CRISPR/Cas9 system. NIS is a transmembrane protein that enables imaging and identification of transplanted cells using radiotracers. When cardiomyocytes derived from these cells (NIS-RhiPSC-CM) were injected into MI-induced mice, these cells could be detected even 8–10 weeks after transplantation [133]. Although, further evaluation of cardiac function was not conducted in this research. However, this is meaningful in that the CRISPR/Cas9 system has been used to find a way to detect myocardial cells from iPSCs in cardiovascular disease models. The therapeutic strategy of knocking out genes through GE has also been attempted. HLA light chain beta-2 microglobulin (B2M) is a molecule belonging to HLA class I and is known to be associated with immune rejection [134]. Since most stem cells have the HLA-1 antigen, CD8+ T cells recognize and attack the transplanted stem cells [135]. For this reason, genetic deletion of B2M gene from human umbilical MSCs utilizing CRISPR/Cas9, reduces the immune rejection and ensure stable engraftment. When UMSCs with knocked out B2M was transplanted into a rat ischemic disease model, the cell death induced by CD8+ T cells was reduced and cardiac fibrosis was also suppressed through exosomes. Eventually, recovery effects of impaired cardiac function were noted, and it is assumed that there was a change in exosome imprinting at the base [136]. The strategy using these cells is a seemingly new breakthrough in overcoming immune rejection, and in myocardial tissue repair and regeneration.

### Therapeutic Application of IL-10 Induced Stem Cells through Various Genome-Engineering Systems

**Fig. 3:**
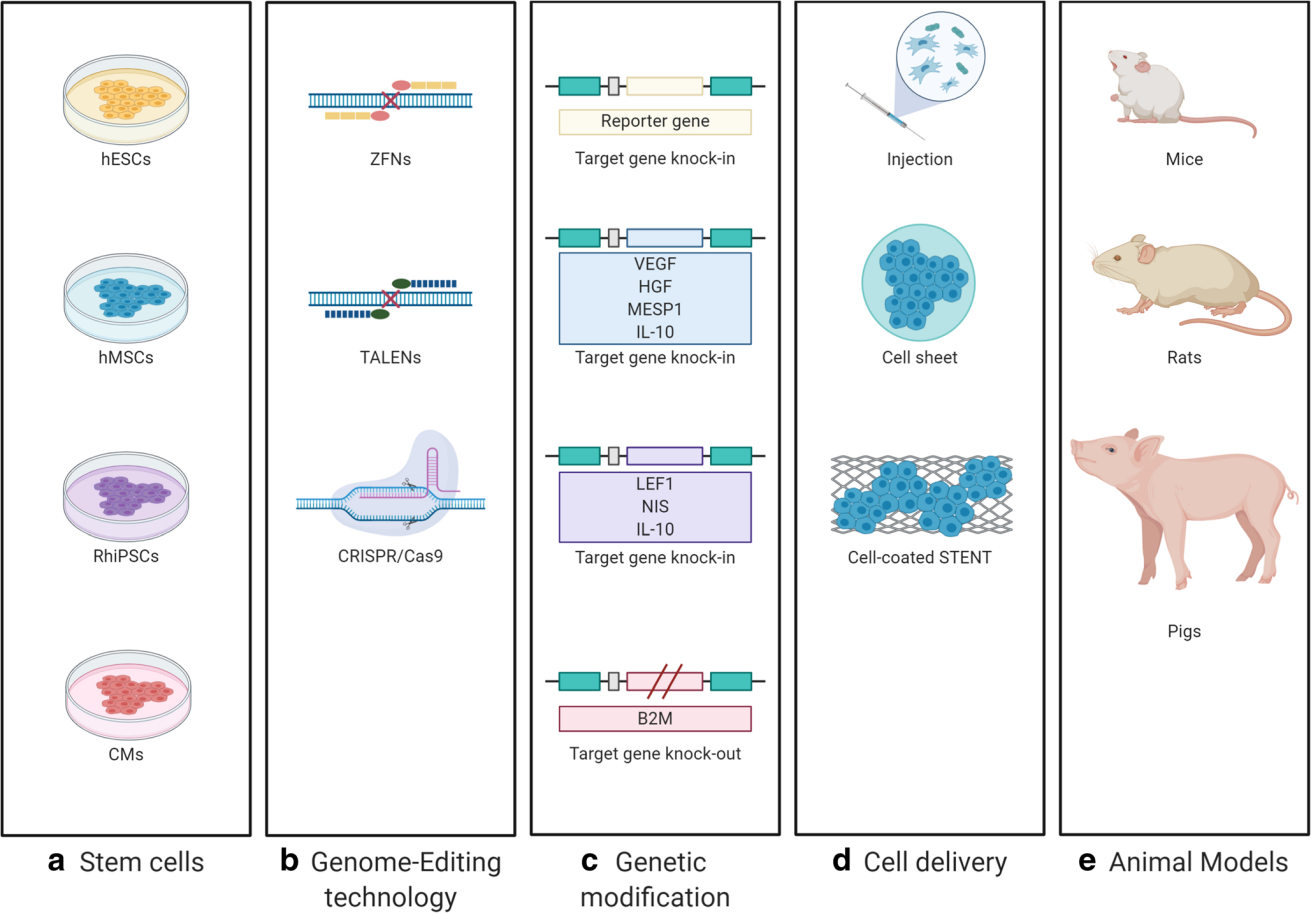
Applications of the genome-edited stem cells in myocardial infarction models. **a** Stem cells used for genome-editing. **b** Genome-editing technologies: ZFNs, TALENs, CRISPR/Cas9 system. **c** Targeted genetic modifications in each stem cells. **d** Cell delivery methods utilized for adaption to animal models. **e** Preclinical models with genome-edited stem cells applied

Trials have been conducted that genetically integrate IL-10 into many stem cells using various GE technologies including TALEN, CRISPR/Cas9, etc. IL-10 is an anti-inflammatory cytokine that inactivates immune responses to pathogens. In the mouse model of MI, treatment with recombinant IL-10 suppressed the inflammatory response and reduced fibrosis by suppressing the expression of MMP-9. In addition, it enhanced the capillary density in the ischemic region by STAT3 activation. Endeavoring to utilize these therapeutic mechanisms of IL-10, a study that edited IL-10 into amniotic MSCs using TALEN and a study that overexpressed this gene in bone marrow derived MSCs using CRISPR activation were conducted respectively. In both studies the produced cells were transplanted into a mouse ischemic heart disease model, and a reduction of pro-inflammatory factors was noted, thereby, reducing the myocardial cell apoptosis and enhancing the capillary density. As a result, it was possible to confirm the cardiac function recovery in cardiovascular disease models [137, 138] (Fig. 3).

## Conclusions

Myocardial cell death is a major concern in ischemic heart disease and reperfusion, since it can be directly linked to the deterioration of cardiac function. As the overall understanding of cell death has improved, not only has it become possible to explain major death mechanisms such as apoptosis, necrosis, autophagy, etc. in myocardial cells, but various treatments targeting them have also been able to be examined. Although, treatment with stem cells has already proven its effectiveness in many animal models, the results of most clinical trials using it have not been very positive. However, the potential of stem cells remains, and genome engineering technology, which has continued to develop over recent years, has enabled stem cells to generate stable and safe genetic modifications. These techniques have been implemented in stem cell therapy and their therapeutic effects have been demonstrated in various animal disease models. Genome engineered stem cell therapy is a promising therapeutic strategy for ischemic heart disease and reperfusion that can overcome the limitations of conventional stem cell therapy.
